# Stochastic epigenetic mutations as possible explanation for phenotypical discordance among twins with congenital hypothyroidism

**DOI:** 10.1007/s40618-022-01915-2

**Published:** 2022-09-07

**Authors:** D. Gentilini, M. Muzza, T. de Filippis, M. C. Vigone, G. Weber, L. Calzari, A. Cassio, M. Di Frenna, M. Bartolucci, E. S. Grassi, E. Carbone, A. Olivieri, L. Persani

**Affiliations:** 1 Bioinformatics and Statistical Genomics Unit, Istituto Auxologico Italiano IRCCS, Cusano Milanino, 20095 Milan, Italy; 2 Department of Brain and Behavioral Sciences, University of Pavia, Pavia, Italy; 3 Laboratory of Endocrine and Metabolic Research, Department of Endocrine and Metabolic Diseases, Istituto Auxologico Italiano IRCCS, Piazzale Brescia 20, 20149 Milan, Italy; 4 Department of Pediatrics, Endocrine Unit, IRCCS San Raffaele Hospital, Milan, Italy; 5 Department of Medical and Surgical Sciences, University of Bologna, Bologna, Italy; 6 Department of Maternal and Child Sciences and Urology, University “La Sapienza”, Rome, Italy; 7 Department of Medical Biotechnology and Experimental Medicine, University of Milan, 20122 Milan, Italy; 8 Department of Cardiovascular and Endocrine-Metabolic Diseases and Aging, Italian National Institute of Health, 00161 Rome, Italy

**Keywords:** Thyroid, Genome-wide DNA methylation, Congenital diseases, Preterm delivery, Twin gestation, Thyroid dysgenesis

## Abstract

**Purpose:**

The elevated frequency of discordance for congenital hypothyroidism (CH) phenotype between monozygotic twins suggests the involvement of non-mendelian mechanisms. The aim of the study was to investigate the role of epigenetics in CH pathogenesis.

**Methods:**

A genome-wide DNA methylation analysis was performed on the peripheral blood of 23 twin pairs (10 monozygotic and 13 dizygotic), 4 concordant and 19 discordant pairs for CH at birth.

**Results:**

Differential methylation analysis did not show significant differences in methylation levels between CH cases and controls, but a different methylation status of several genes may explain the CH discordance of a monozygotic twin couple carrying a monoallelic nonsense mutation of *DUOX2*. In addition, the median number of hypo-methylated Stochastic Epigenetic Mutations (SEMs) resulted significantly increased in cases compared to controls. The prioritization analysis for CH performed on the genes epimutated exclusively in the cases identified *SLC26A4, FOXI1, NKX2-5* and *TSHB* as the genes with the highest score. The analysis of significantly SEMs-enriched regions led to the identification of two genes (*FAM50B* and *MEG8*) that resulted epigenetically dysregulated in cases.

**Conclusion:**

Epigenetic modifications may potentially account for CH pathogenesis and explain discordance among monozygotic twins.

**Supplementary Information:**

The online version contains supplementary material available at 10.1007/s40618-022-01915-2.

## Introduction

Congenital hypothyroidism (CH) is the most common congenital endocrine disease and an avoidable cause of severe mental retardation [1].

The CH pathogenesis may include the contribution of genetic and environmental factors [1–3]. Nevertheless, the pathogenic mechanisms of CH are still largely undefined, suggesting the involvement of unidentified genes or alternative mechanisms, also supported by the elevated frequency of discordance for CH phenotype between monozygotic twins [4–6].

Non-Mendelian mechanisms include epigenetic modifications that can produce phenotype changes without gene sequence variations but involve alterations in gene transcription [7]. In particular, DNA methylation is an epigenetic modification in which methyl groups are added to the cytosine residues within CpG dinucleotides, thereby preventing the binding of transcription factors to DNA. Interestingly, premature birth, which represents a relevant risk factor for CH, was previously associated with alterations in DNA methylation [8–10].

To date, systematic methylome analysis in CH has been performed only in the context of thyroid ectopy [11, 12]. However, no differences in methylation profile have been found between ectopic and orthotopic thyroid tissues [11] and between peripheral leucocytes of CH cases with ectopy compared to normal controls [12].

The aim of this study was to investigate the role of epigenetics in CH pathogenesis. To reach this goal we performed a genome-wide DNA methylation analysis in the peripheral whole blood from a large cohort of CH twins, 23 twin pairs (10 monozygotic and 13 dizygotic), of whom 4 concordant and 19 discordant for CH at birth.

## Materials and methods

### Study design/population

CH cases were enrolled in several Italian referral centers within a specific research protocol that was approved by the Ethics Committees of the involved institutions. The sample size was calculated considering a methylation difference between groups of at least 7% and a power of 80%, as previously reported [13].

### Genetic analysis

Genetic analyses were performed on both affected and unaffected twins by NGS of a panel including 11 CH candidate genes, as previously reported [14].

### Illumina humanmethylation450K BeadChip array

Array-based procedure was carried out following the manufacturer’s instructions and using Illumina-supplied reagents and conditions as described [15] after bisulfite conversion of genomic DNA.

### Differential methylation analysis

Paired differential methylation analysis between case and control groups was performed using the RnBeads (2.4.0) package [16] in R environment (version 3.6.1).

### Stochastic epigenetic mutations (SEMs)

Stochastic Epigenetic Mutations (SEMs) and regions enriched in SEMs were identified as previously described by our group [10, 15, 17–19].

## Statistics

Statistical analyses were performed in R package, as reported in Supplemental Methods.

## Results

### Clinical data and genetic analysis

Ten MZ and 13 DZ pairs of twins were enrolled (Tables 1 and 2). Eight pairs, 2 MZ (#1A and B, #2A and B) and 2 DZ twins (#11A and B, #12A and B), were concordant for the CH diagnosis. Overall, 27 CH cases and 19 unaffected controls were enrolled. Thyroid dysgenesis was described in 14 cases (5 athyreosis, 1 hemiagenesis, 5 ectopy, 3 hypoplasia) while a gland-in-situ (GIS) of normal or enlarged size was described in 13 cases. The diagnosis at reevaluation was of permanent CH in 19 cases and transient in 8 cases, all cases with thyroid dysgenesis and 5/13 (38%) with GIS resulted permanent. One discordant MZ twin (#9B) at neonatal screening was confirmed as euthyroid at 10 years and diagnosed with hypothyroidism at 12 years.

**Table 1:** Clinical and molecular characteristics of monozygotic (MZ) twins

#	Gender	bsTSH @ SCR mU/l	sTSH @ D mU/l	FT4 @ D ng/dl	US @ D	Age @ sampling	Final diagnosis @ RE	Mutations	GA	Hypo SEMs
**1A**	**F**	**–**	**7.6**	**1.11**	**GIS**	**4 years** **7 months**	**P**	***DUOXA2* p.W4R (PA)**	**24.3**	**9967**
**1B**	**F**	**–**	**8.0**	**1.06**	**GIS**	**4 years** **7 months**	**P**	***DUOXA2* p.W4R (PA)**	**24.3**	**8284**
**2A**	**M**	**8.35**	**12.6**	**–**	**GIS**	**3 years** **2 months**	**T**	**WT**	**34.3**	**6830**
**2B**	**M**	**8.0**	**14.8**	**–**	**GIS**	**3 years** **2 months**	**T**	**WT**	**34.3**	**3548**
**3A**	**F**	**31**	**234**	**0.3**	**Athyreosis**	**14 years** **5 months**	**P**	**WT**	**38**	**1366**
3B	F	1.0	1.1	1.2	GIS	14 years 5 months	E	WT	38	1980
**4A**	**M**	**200**	**380**	**0.3**	**Athyreosis**	**21 years**	**P**	**WT**	**36**	**2642**
4B	M	1.2	2.0	–	GIS	21 years	E	WT	36	2122
**5A**	**F**	**8.2**	**15.55**	**1.1**	**GIS**	**5 years** **7 months**	**T**	**WT**	**27.5**	**1608**
5B	F	3.0	4.8	1.3	GIS	5 years 7 months	E	WT	27.5	1233
**6A**	**F**	**104**	**574**	**0.31**	**Sublingual ectopy**	**3 years** **4 months**	**P**	***SLC26A4*: p.T410M (PA), p.V678V (V)**	**41**	**2202**
6B	F	2.4	6.5	1.64	GIS	3 years 4 months	E	*SLC26A4*: p.T410M (PA), p.V678V (V)	41	2191
**7A**	**F**	**199**	**199**	**0.1**	**Athyreosis**	**3 years**	**P**	**WT**	**38**	**2633**
7B	F	1.1	3.0	0.9	GIS	3 years	E	WT	38	3475
**8A**	**F**	**395**	**395**	**0.7**	**Athyreosis**	**11 years** **1 months**	**P**	***DUOX2* p.Q556X (PA)**	**38.5**	**11,772**
8B	F	1.5	2.0	0.7	GIS	11 years 1 months	E	*DUOX2* p.Q556X (PA)	38.5	2255
**9A**	**F**	**49**	**228**	**0.11**	**Ectopy**	**10 years**	**P**	***GLIS3* p.G313A (PA)**	**35.2**	**1773**
9B	F	3.5	–	0.71	GIS	10 years	P	*GLIS3* p.G313A (PA)	35.2	2053
**10A**	**F**	**84**	**147**	**0.5**	**Ectopy**	**2 years** **9 months**	**P**	**WT**	**34**	**3002**
10B	F	1.0	6.1	–	GIS	2 years 9 months	E	WT	34	3227

The rows in bold indicate CH case

*SCR* screening, *D* diagnosis, *RE* re-evaluation, *GA* gestational age, *hypo-SEM* hypo-methylated Stochastic Epi-genetic Mutations, *–* not available, *GIS* gland-in situ, *y* year, *m* month, *T* transient, *P* permanent, *E* euthyroid, *PA* pathogenic, *B* benign, *V* variant of uncertain significance

**Table 2:** Clinical and molecular characteristics of dizygotic (DZ) twins

#	Gender	bsTSH @ SCR mU/l	sTSH @ D mU/l	FT4 @ D ng/dl	US @ D	Age @ sampling	Final diagnosis @ RE	Mutations	GA	Hypo SEMs
**11A**	**M**	**8.4**^**a**^	**9.25**	**0.98**	**hypoplasia**	**6 years** **2 months**	**P**	**WT**	**32.5**	**21,528**
**11B**	**M**	**9.2**^**a**^	**7.7**	**1.18**	**hypoplasia**	**6 years** **2 months**	**P**	**WT**	**32.5**	**23,996**
**12A**	**M**	**14**	**15.3**	**1.3**	**emiagenesis**	**2 years** **9 months**	**P**	**WT**	**38**	**1665**
**12B**	**M**	**8.2**	**9.3**	**1.26**	**GIS**	**2 years** **9 months**	**P**	**WT**	**38**	**2057**
**13A**	**M**	**13.2**	**39.4**	**1.3**	**GIS**	**1 years** **5 months**	**P**	***NKX2-1* p.A116D (PA)**	**37.4**	**10,399**
13B	M	1.0	4.6	1.61	GIS	1 years 5 months	E	WT	37.4	6663
**14A**	**M**	**11**	**150**	**0.3**	**GIS**	**7 years** **6 months**	**T**	**WT**	**37.3**	**1712**
14B	F	< 10	3.7	1.28	GIS	7 years 6 months	E	WT	37.3	1987
**15A**	**F**	**520**	**1800**	**0.1**	**Ectopy**	**20 years**	**P**	**WT**	**36**	**3433**
15B	M	1.0	2.7	–	GIS	20 years	E	WT	36	2288
**16A**	**F**	**257**	**943**	**0.2**	**athyreosis**	**14 years**	**P**	**WT**	**35.5**	**1771**
16B	F	1.0	1	–	GIS	14 years	E	WT	35.5	1821
**17A**	**M**	**19.5**	**137**	**0.54**	**GIS**	**10 years** **10 months**	**T**	**WT**	**37.5**	**2649**
17B	M	0.8	1	–	GIS	10 years 10 months	E	WT	37.5	3463
**18A**	**F**	**8.1 **^**a**^	**11**	**0.9**	**GIS**	**3 years**	**T**	***DUOX2* p.A728T (B/PA functional study);** ***SLC26A4* IVS6 + 4 bp *A* > *C* (V);** ***PAX8* p.K135R (PA)**	**28.5**	**3388**
18B	F	4.3	5.0	1.1	GIS	3 years	E	WT	28.5	2681
**19A**	**F**	**38**	**18**	**1.25**	**GIS**	**3 years** **7 months**	**T**	***GLIS3* p.E515D (B)**	**36.6**	**3163**
19B	M	1.0	1.0	–	GIS	3 years 7 months	E	WT	36.6	1599
**20A**	**F**	**15**	**58**	**1.23**	**GIS**	**4 years** **10 months**	**T**	***DUOX2* p.E641K (B)**	**38.5**	**6806**
20B	F	4.0	2.4	0.98	GIS	4 years 10 months	E	WT	38.5	2545
**21A**	**F**	**130**	**333**	**0.5**	**Ectopy**	**14 years**	**P**	***DUOX2* p.R726W (PA);** ***GLIS3* p.P376S (B), p.P364S (V)**	**34.6**	**3362**
21B	F	1.2	3.0	–	GIS	14 years	E	*GLIS3* p.P376S (B)	34.6	2205
**22A**	**M**	**8.4**^**a**^	**10**	**1.02**	**GIS**	**6 years**	**P**	**WT**	**32.4**	**1453**
22B	M	1.1	–	–	GIS	6 years	E	*TPO* p.A419E (B)	32.4	1213
**23A**	**F**	**25**	**39.3**	**1.87**	**hypoplasia**	**2 years** **8 months**	**P**	***TPO* p.P135H (PA/B Clin Var);** ***SLC26A4* p.I455F (V)**	**37**	**3446**
23B	F	3.4	4.5	2.31	GIS	2 years 8 months	E	WT	37	3638

The rows in bold indicate CH case

*SCR* screening, *D* diagnosis, *RE* re-evaluation, *GA* gestational age, *hypo-SEM* hypo-methylated Stochastic Epi-genetic Mutations, *–* not available, *GIS* gland-in-situ, *y* year, *m* month, *T* transient, *P* permanent, *E* euthyroid, *PA* pathogenic, *B* benign, *V* variant of uncertain significance

^a^In preterm infants, TSH cutoff @ second screening is 5.0 mU/L (31)

Genetic analysis was performed in all CH patients and healthy co-twins. In particular, 41% (11/27) of CH children resulted to carry at least one variant in one of the 11 candidate genes.  While no difference in genetic data were seen among all the MZ twin couples, rare variants in the candidate genes were detected only in CH cases from 6/11 discordant DZ twin couples.

### Immunological characteristic of subjects

Blood cell counts have been estimated from methylation data. The mixed effect regression model considering family as random effect and sex and batch effect as potential confounders failed to identify significant differences between the two groups (Fig. S2).

### DNA methylation profiling using multi-dimensional scaling (MDS)

**Fig. 1:**
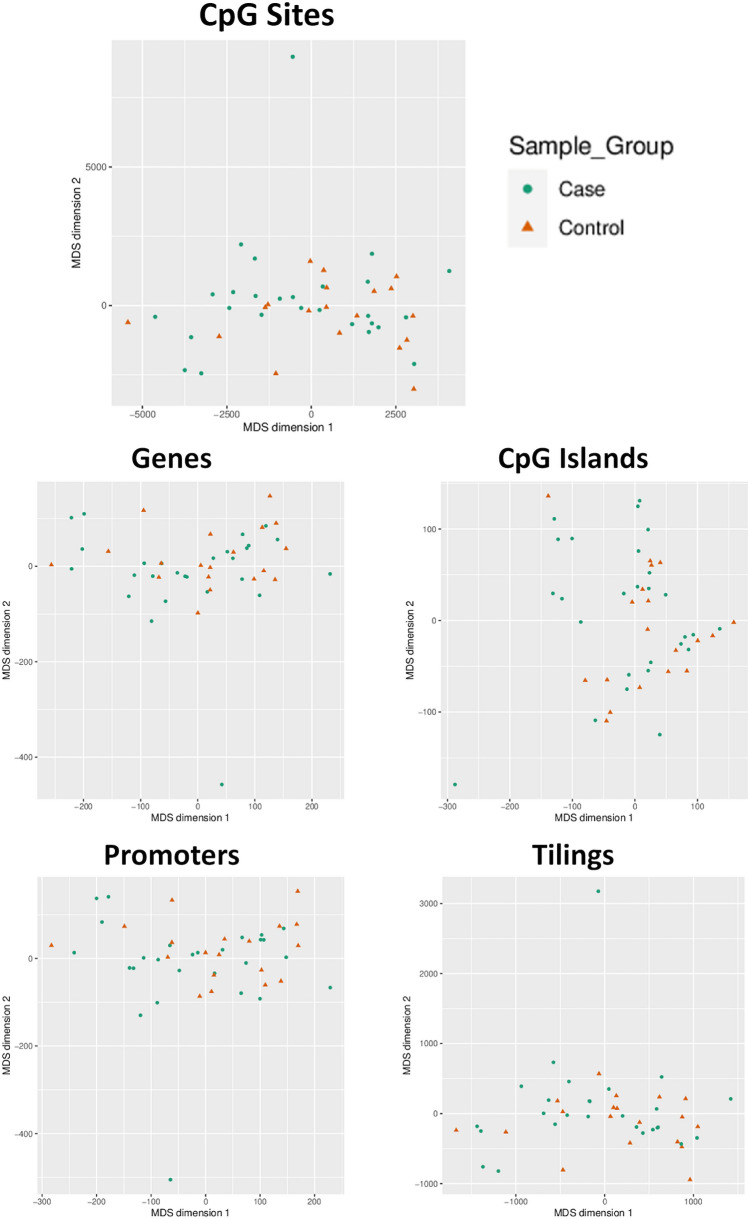
Dimensional reduction of methylation data. Scatter plots show samples after performing Kruskal's non-metric multidimensional scaling. Only the first two dimensions are shown. Subjects are represented according to the Sample Group variable

Dimensional reduction was used to visually inspect the dataset for strong signals in the methylation values. The MDS was adopted considering methylation signals from sites and considering genomic regions: Genes, CpG islands, Promoters and Tilings. No macroscopic differences were observed in the methylation profile between cases and controls considering sites or genomic regions (Fig. 1).

Similarly, we did not observe noticeable differences also taking into account the zygosity status, the presence of a genetic variant, the family origin or the thyroid morphology (Dysgenesis or GIS) (Fig. S3A–D).

### Differential methylation analysis

**Fig. 2:**
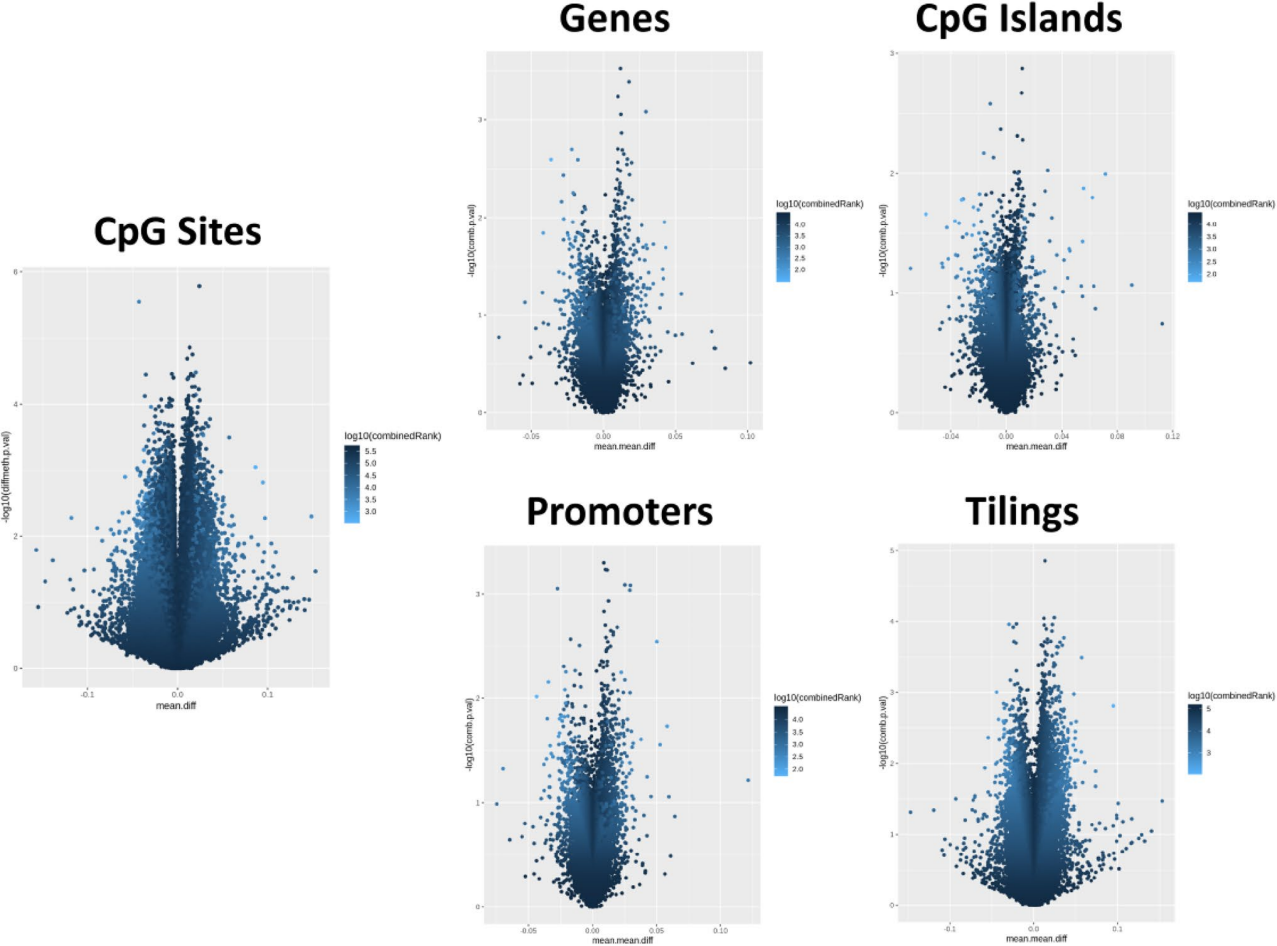
Volcano plots representing paired differential methylation analysis. Analysis was carried out at site (left panel) and at regional level (right panels). Non-adjusted *p* values, expressed as – Log10 transformed values, are represented in the *Y*-axis while differences in methylation levels in the *X*-axis

Paired differential methylation analysis was computed both at site and at region level and results were represented as volcano Plot (Fig. 2). At site level, after False Discovery Rate adjustment, no statistical differences in methylation between cases and controls emerged. A list of the top 50 ranked differently methylated regions is reported in Table S1. Differential methylation analysis performed at regional level was conducted considering genes, CpG islands, promoters and tilings. After multiple testing correction, no statistical differences in region methylation levels emerged between cases and controls. A list of the top 50 ranked differently methylated regions is reported in Table S2.

### Gene ontology and functional analysis

To focus the analysis on a more consistent list of markers, a Gene Ontology (GO) enrichment analysis was performed considering the list of genes and promoters found to be significant, at least, at nominal level (unadjusted *p* value < 0.05). GO enrichment on genes (Fig. S4) highlighted some biological processes, mainly concerning Olfactory Perception and G protein-coupled receptor signaling pathways. About promoter regions, analysis confirmed the same biological functions observed for gene classification (data not shown).

### Stochastic epigenetic mutations (SEMs) analysis

**Fig. 3:**
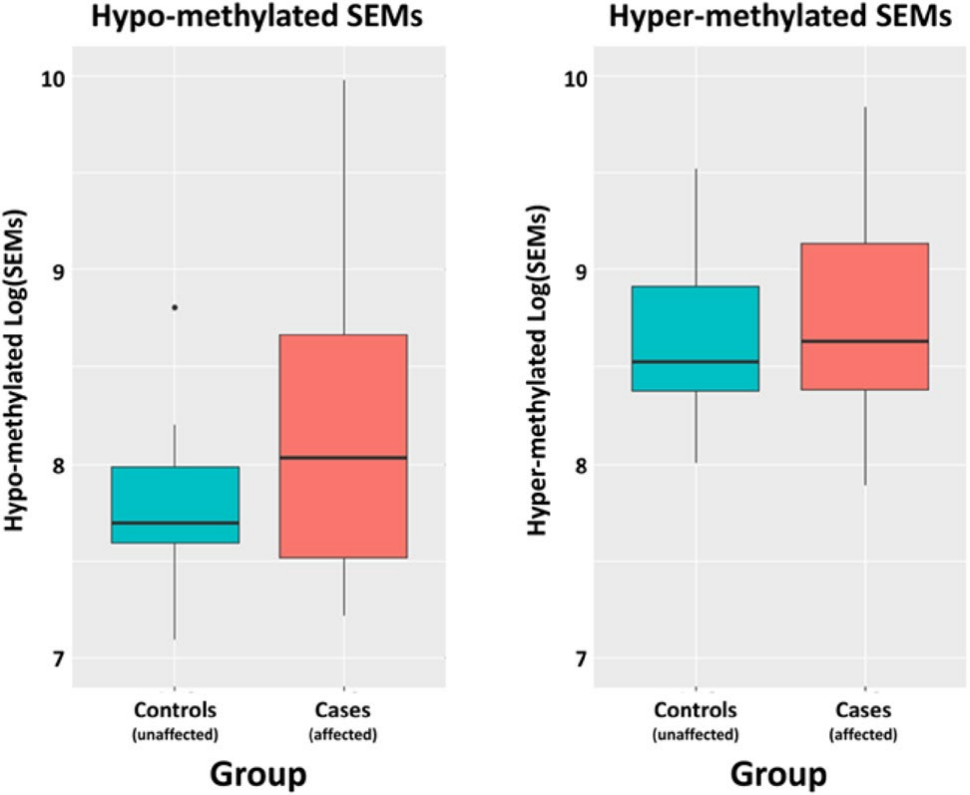
Boxplot showing the number and of hypo- and hyper-methylated log(SEMs). The thick horizontal line represents the median of the distribution while the box represents the interquartile range. Whiskers are set as the default option for the “boxplot” function and show the most extreme data point, which is no more than 1.5 times the interquartile range from the box. Dots represent outlier values (single values exceeding 1.5 interquartile ranges)

The median number of hypo-methylated SEMSs (ln(SEMs)) was 8.059 (*Q*1 = 7.555; *Q*3 = 8.827) in the affected group and 7.698 (*Q*1 = 7.593; *Q*3 = 7.987) in the control group. The mixed effect regression model considering “sex” variable as potential confounder and “family” as random effect, indicated that this difference was significant (*p* = 0.02). Conversely, the mixed effect regression model showed that hyper-methylated ln(SEMs) resulted not significantly different between the two groups (*p* = 0.50). The median number of hyper-methylated log(SEMs) was 8.632 (*Q*1 = 8.381; *Q*3 = 9.136) in hypothyroid sib-pair and 8.524 (*Q*1 = 8.373; *Q*3 = 8.914) (Fig. 3). The median number of hypo or hyper-methylated SEMs in cases did not correlate with the zygosity (MZ and DZ), the thyroid morphology (dysgenesis or GIS), the CH outcome (permanent vs transient) or the genetic background (data not shown).

### SEMs annotation analysis and candidate markers

**Fig. 4:**
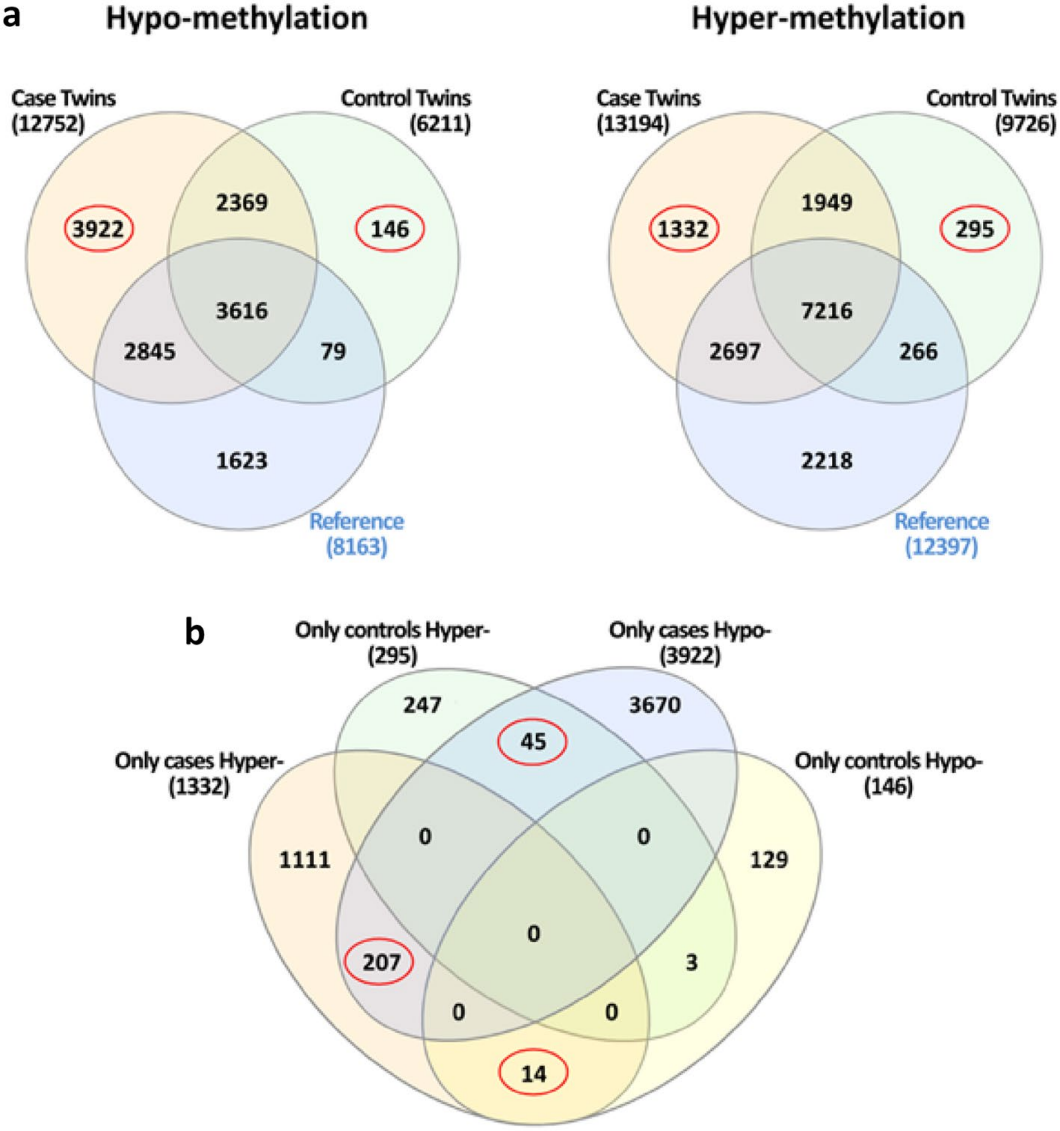
Venn diagrams of **a** genes resulted annotated from hypo- and hyper-methylations in cases, controls, and reference population, and **b** univocal gene lists from cases and controls

Both for hyper- and hypo-methylated SEMs we annotated genomic positions and obtained the name of genes involved. Based on gene annotations, we selected loci that resulted epimutated only in cases or in controls. The Venn diagram in Fig. 4 describes the strategy adopted to select these genes.

Through this procedure, concerning hypo-methylations, two lists of 3922 and 146 genes, univocally belonging to the case and control groups were identified, respectively. Similarly, with regards to hyper-methylations, 1332 and 295 univocal genes emerged from Venn analysis (Fig. 4a). The list of these genes is reported in Table S3. As a further refinement, univocal gene lists were also investigated: the Venn diagram in Fig. 4b shows that analysis identified two common sets of genes with discordant SEMs methylation profile (*n* = 45 for univocal controls hyper- vs. univocal cases hypo-methylated; *n* = 14 for univocal cases hyper- vs. univocal controls hypo-methylated). Moreover, a list of 207 genes has been found to be associated to both hyper- and hypo-methylations in cases.

### Gene ontology—prioritization analysis

A gene ontology analysis was performed on genes found univocally epimutated in the case group: analysis showed “regulation of protein acetylation” and “organic hydroxy compound metabolic process” as the most enriched pathways in hyper- or hypo-methylated genes, respectively (Fig S5). The prioritization analysis considering hypo-thyroidism as a unique disease term on the genes that resulted epimutated only in cases identified *SLC26A4*, *NKX2-5*, *TSHB*, and *FOXI1* as the gene with the highest score in hypo-methylated group and *SLC26A4*, and *FOXI1* in hyper-methylated group (Table S4).

### SEM-enriched region analysis

**Fig. 5:**
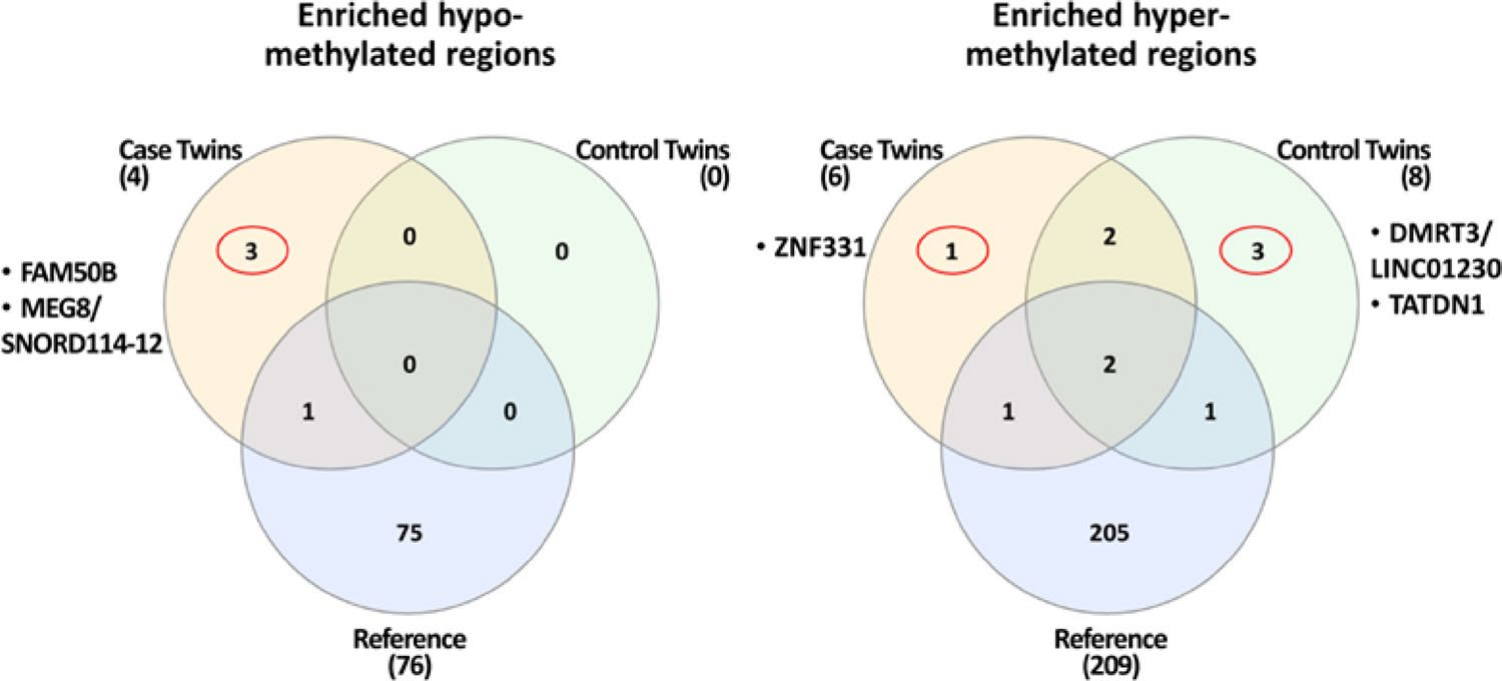
Venn diagrams of SEMs-enriched genes in CH cases, controls and reference population: hypo-methylations (left panel) and hyper-methylations (right panel)

For each subject, genomic coordinates of SEM-enriched regions were reported and annotated to obtain the list of genes involved. Finally, the three populations were compared through a Venn diagram to identify cases’ or controls’ specific epigenetic alterations (Fig. 5).

**Fig. 6:**
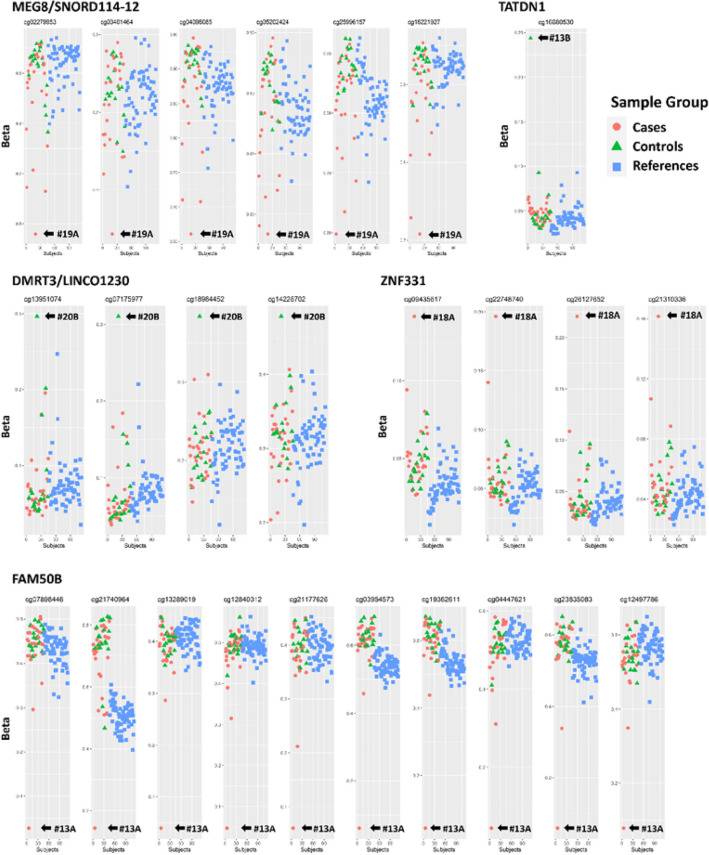
Scatter plots of cases/controls/references methylation profiles for *MEG8/SNORD114-12*, *TATDN1* (upper-panel), *DMRT3/LINC01230, ZNF331* (middle-panel) and *FAM50B* (lower-panel) genes. The arrows identify SEMs in the involved subject

The analysis identified 4 SEM-enriched genes present only in cases (3 hypo-methylated and 1 hyper-methylated) and 3 SEM-enriched genes present only in controls (all hyper-methylated). Hypo-methylated SEM involved three genes: *FAM50B* (#13A), *MEG8* and *SNORD114-12* (#19A) while hyper-methylated SEMs involved four genes: *ZNF311* (#18A), *TATDN1* (#13B), *DMRT3* and *LINC01230* (#20B). For these genes, the methylation levels of SEMs have been represented (Fig. 6).

## Discussion

The role of epigenetics in the aetiology of CH has been scantly investigated so far. Here, we compared, for the first time, circulating DNA methylome profiles of both MZ and DZ twin pairs concordant or discordant for CH diagnosis.

The absence of a significant DNA methylation signatures at genome-wide level in CH cases may likely indicate a broad epigenetic heterogeneity in this rare condition.

Stochastic Epigenetic Mutations (SEMs) represent a potent biomarker of epigenetic drift and an effective indicator of exposure-related accumulation of DNA damages [17]. Recently, rare epigenetic mutations were found significantly enriched in cases with congenital anomalies and were associated with altered gene expression [20]. Interestingly, we identified a significant increase of hypo-methylated SEMs in hypothyroid twin pairs compared to the healthy-twins cohort. This finding suggests that thyroid defects could be associated with an increased expression of predisposing genes in CH-affected twins [20, 21]. Epigenetic drift can be defined as the accumulation of mistakes in maintaining normal epigenetic patterns. This process contributes to impaired cellular and molecular functions and to a decline in phenotypic plasticity at the cellular and molecular levels [22]. Garg et al. (2020) [21] found that approximately one-third of epivariations are discordant between MZ twins, indicating that a significant fraction of epivariations occurs post-zygotically. These epigenetic marks are thought to be particularly vulnerable to environmental stressors in the perinatal period and are maintained across different cell lineages [23]. The involvement of these epivariations may account for the different phenotypes observed in twins, independently from the outcome of CH (permanent vs transient).

The prioritization analysis for CH performed on the genes epimutated only in cases identified *SLC26A4*, *FOXI1* (both hypo and hyper-methylated), *NKX2-5* and *TSHB* (hypo-methylated) as the genes with the highest score. Biallelic mutations in *SLC26A4* gene cause Pendred syndrome, characterized by sensorineural hearing loss, enlarged vestibular aqueduct, goiter, and variable CH. FOXI1 is an upstream regulator of *SLC26A4* transcription; monoallelic mutations of *FOXI1* were documented in patients with sensorineural hearing loss and inner ear malformations [24]. The *NKX2-5* gene is a member of the homeobox Nkx2 family that has been implicated in the pathogenesis of CH [25]. *TSHB* encodes the beta subunit of thyroid-stimulating hormone and a reduced methylation status at this level might favor the circulating TSH rise in CH patients and potentially explain the relative pituitary refractoriness in the normalization of circulating TSH occurring in some patients with CH during levothyroxine replacement [26–29].

Moreover, an in-deep analysis of single-pairs twins revealed interesting findings.

Pair #8 consists of MZ twins discordant for CH. The #8A presents CH and athyreosis. The genetic analysis revealed that both twins carried a nonsense mutation in *DUOX2* gene (p.Q556X). Noteworthy, the phenotypical discordance among these two monozygotic twins argues against the possibility that monoallelic *DUOX2* variants can be sufficient to explain the appearance of CH in one family [30]. Previous works showed mutations in genes typically associated with functional defects, including *DUOX2*, that had been also detected in thyroid dysgenesis, a finding frequently justified by the association with other genetic events in the oligogenic model of CH [14]. However, an additional occult genetic event explaining the discordance for thyroid dysgenesis and CH among two monozygotic twins is at least unlikely. Intriguingly, the #8A affected twin presented several genes with a significant differential hypo-methylation compared to the unaffected twin (Tables 1 and S5), which may instead explain the discordant phenotypical presentation of these MZ twins despite the shared heterozygous *DUOX2* variation. Among the differentially methylated genes detected in this couple, the *BICC1* gene might be relevant in this context (Table S5). *BICC1* encodes an RNA-binding protein that is active in regulating gene expression during embryonic development and involved in stress responses to maintain tissue and organ integrity [31].

Pair #11 consists of DZ concordant twins presenting thyroid hypoplasia and permanent CH, without pathogenic variants in the analyzed genes. Interestingly, the burden of epimutations in this couple was the highest detected (Tables 2 and S5). These twins showed epimutations in most of the genes prioritized for CH phenotype (Tables S4 and S5).

Pair #12 consists of DZ concordant twins with permanent CH without pathogenic variants in the analyzed genes. Both presented thyroid dysgenesis, but the #12A with thyroid hemiagenesis and the #12B with hypoplasia. Of note, #12A showed an epimutation in *SLC26A4* gene (Table S5). Interestingly, despite being classically implicated in thyroid hormonogenesis, *SLC26A4* variations were reported also in patients with apparent thyroid dysgenesis [32]. Of note, this patient does not present any hearing impairment.

Case #13A was found to be a carrier of a benign *NKX2-1* heterozygous variant (p.A116D) lacking the typical extrathyroidal manifestations of Brain-Lung-Thyroid syndrome [33]. Interestingly, this case showed a hypo-methylated SEM-enriched region in *FAM50B* gene (Family with Sequence Similarity 50 Member B, 6p25.2), which encodes a protein that plays a role in the circadian clock. *FAM50B* is an imprinted gene paternally expressed in many tissues, including the thyroid gland (https://gtexportal.org). Hypo-methylation of the same region of *FAM50B* has been previously reported in a patient with development delay [34], and has been associated with multi-locus imprinting defect (MLID) [35]. *FAM50B* hypo-methylation could have contributed to the appearance of the CH in association with a heterozygous *NKX2-1* variant not sufficient per se to explain the phenotype in this case.

Case #19A is a DZ twin discordant for CH, with GIS and a heterozygous benign variation in *GLIS3* in the absence of the typical manifestation of neonatal diabetes [27]. This case showed a hypo-methylated SEM-enriched region on chromosome 14q involves *MEG8* (Maternally Expressed 8, Small Nucleolar RNA Host Gene) and *SNORD114-12* (Small Nucleolar RNA, C/D Box 114-12), two RNA genes, located in an imprinted locus containing differentially methylated regions (IG-DMR, MEG3-DMR, MEG8-DMR), slightly expressed in the thyroid gland (https://gtexportal.org). A hypo-methylated status in these regions is responsible for Temple and Kagami-Ogata syndromes [35, 36] and these were identified in two unrelated cases with neurodevelopmental disorders and congenital anomalies [20]. The severe hypo-methylation status of a different region of *MEG8* (Int29-32), potentially resulting in the *MEG8* overexpression, could explain the appearance of the isolated CH phenotype in this case.

This study presents some limitations. In the first place, the lack of significant differences in global methylation between affected and unaffected twins may be a consequence of the limited sample cohort. In addition, our analysis was performed on blood samples collected later in life during levothyroxine replacement in CH cases. Nevertheless, a recent study confirmed that epivariations are conserved across multiple tissues, validating the use of peripheral blood for epigenomic analyses [20]. The effect of replacement therapy may instead have mitigated some differences between cases and controls. Finally, these determinations require replications in independent cohorts.

In conclusion, epigenetic modifications may be included among the possible mechanisms that, possibly in association with other events (e.g., hypomorphic thyroid alleles), can account for CH pathogenesis and discordance among monozygotic twins. Their relevance may be particularly high in conditions characterized by an increased risk for CH such as premature birth or low birth weight.

## Supplementary Information

Below is the link to the electronic supplementary material.

Supplementary file1 (DOCX 42 KB)

Supplementary file2 (DOCX 8900 KB)

Supplemental Table S1 List of the top 50 ranked differently methylated CpG sites obtained by the RnBeads Differential Methylation Analysis Supplementary file3 (XLSX 24 KB)

Supplemental Table S2 List of the top 50 ranked differently methylated regions (Genes, Promoters, CpG Islands, Tiling) obtained by the RnBeads Differential Methylation Analysis Supplementary file4 (XLSX 47 KB)

Supplemental Table S3 List of univocal genes obtained by comparing hypo- or hyper-methylated SEMs of cases vs controls Supplementary file5 (XLSX 85 KB)

Supplemental Table S4 Results of prioritization analyses carried out considering hypothyroidism as a unique disease term on the genes that resulted “hypo-epimutated” or “hyper-epimutated” only in cases Supplementary file6 (XLSX 14 KB)

Supplemental Table S5 Number of SEMs vs annotated genes (cases, controls and references) Supplementary file7 (XLSX 3170 KB)

## Data Availability

All data are deposited to GEO (https://www.ncbi.nlm.nih.gov/geo/) with accession number GSE161041.
