# Supplementary material for: Stochastic epigenetic mutations as possible explanation for phenotypical discordance among twins with congenital hypothyroidism
Source: J Endocrinol Invest. 2022 Sep 7;46(2):393–404. doi: 10.1007/s40618-022-01915-2 (PMC9859866; doi:10.1007/s40618-022-01915-2)
Supplement: Supplementary file 1 — Supplementary file1 (DOCX 42 KB) [file 40618_2022_1915_MOESM1_ESM.docx]

**Supplemental methods**

***Inclusion criteria***

Inclusion criteria were: twin delivery and positive neonatal TSH screening (dried blood spot TSH) with a diagnosis of primary CH confirmed by serum thyroid function tests at 1-3 weeks of age following the previously illustrated criteria [[1](#_ENREF_1), [2](#_ENREF_2)]. The analysis was applied to the first 23 eligible twin pairs, either monozygotic (MZ) or dizygotic (DZ), concordant or discordant for CH diagnosis at birth, enrolled after the approval of the protocol.

***Genetic analysis***

Genetic analyses were performed by NGS of a panel including 11 CH candidate genes (DUOX2, DUOXA2, FOXE1, GLIS3, JAG1, NKX2-1, PAX8, SLC26A4, TG, TPO, TSHR). All regions not correctly sequenced were recovered by Nextera® DNA Library Preparation kit (Illumina, San Diego, CA). Basic data analysis was performed according to the default parameters of the Illumina’s MiSeq Reporter software. The functional consequences of genetic variations were annotated with wANNOVAR tool [[3](#_ENREF_3)]. All variants of interest were verified by conventional dideoxy sequencing using BigDye® Terminator v.3.1 Cycle Sequencing Kit (Life Technologies, Carlsbad, CA, USA) on a 3100 DNA Analyzer from Applied Biosystems (Foster City, CA). The variants with the minor allele frequency (MAF) > 0.1% and annotated in public or licensed databases (NCBI-dbSNP, NCBI-CliVar, Ensembl, GnomAD, ExAC Browser, NHLBI GO Exome Sequencing Project and HGMD professional) as benign were excluded by further analysis.

***Assessment of the variant pathogenicity***

Interpretation of the identified variants was performed according to ACMG/AMP 2015 guidelines [[4](#_ENREF_4)] by wIntervar (<http://wintervar.wglab.org/>), a bioinformatics software tool for clinical interpretation of genetic variants [[4](#_ENREF_4)]. We also considered the functional data of the identified variants present in the scientific literature and NCBI:ClinVar reports. For the intronic variants close to the splicing sites (-3/+6 nucleotides from the boundary) we used the following in silico predictive softwares: NetGene2v.2.4, bdgp, ESEfinder2.0. Moreover, we considered as disruptive the nonsense variants.

***Bisulfite conversion***

The gDNA (900 ng) was bisulfite converted by using the EZ DNA Methylation Kit (Ref: D5001, Zymo Research Corporation) according to the manufacturer’s protocol. Specific incubation conditions (Illumina Protocol) were applied. To evaluate conversion efficiency and bisulfite converted DNA (bsDNA) integrity, a single-strand quantification of bsDNA was performed by using NanoPhotometer Pearl (Implen GmbH). Fragmented or too diluted DNA samples were discarded and then reprocessed.

***DNA methylation age and cell types***

DNA methylation ages and proportions of T cells (CD8 naive, CD8, CD4 naïve, CD4), NK cells, B cells, monocytes, and granulocytes were estimated using the Steve Horvath’s DNA Methylation Calculator (<https://dnamage.genetics.ucla.edu/home>) [[5](#_ENREF_5)].

***Differential methylation analysis***

Quality control, pre-processing, and generation of β-values dataset was performed using specific modules. SNP-enriched probes (n=10131), unreliable measurements, context-specific (n=2714), and on sex chromosomes probes (n=11033) were filtered out. As a final outcome of the filtering procedures, 14105 probes and 0 samples were removed. Signal intensities from 458627 probes were normalized using the “dasen” normalization method [[6](#_ENREF_6)]. Paired differential methylation analysis was conducted according to the sample groups by computing p-values through the limma method for the site level analysis. For the analysis of regions (Genes, promoters, CpG island, and tiling) a combined p-value was calculated from the p-values of single sites. The four pre-selected genomic regions are defined as follows: i) genes: Ensembl format genes, version Ensembl Genes 75 (n=30819), ii) Promoters regions: the regions 1.5 kb upstream and 0.5 kb downstream of the transcription start sites (n=30970), iii) CpG Islands: CpG island track of the UCSC Genome Browser (n=26579), and iv) Tiling regions: non-overlapping tiling regions with a fixed window size of 5 kilobases defined over the whole genome (n=135752). To estimate surrogate variables that can account for cell-type composition as well as any other sources of systematic variation/confounders [[7](#_ENREF_7)], a Surrogate Variable Analysis (SVA) was applied in the differential methylation step by using the function directly provided in the RnBeads package. Prioritization of differentially methylated genes was conducted by GO Enrichment Analysis via RnBeads using an algorithm (GOstats) based on a hypergeometric test and the hierarchical structure of the gene ontology database [[8](#_ENREF_8)].

To confirm the zygosity of twin pairs, the degree of genetic similarity was assessed by using the RnBeads quality control module which measures the fluorescence values of the 450K BeadChip genotyping probes. Euclidean distance and complete linkage were used for constructing the dendrograms. As shown in the relative heatmap in Figure S1, the monozygotic/dizygotic state was reasonably confirmed.

***Stochastic Epigenetic Mutations (SEMs)***

The distribution and variability of methylation levels at each single CpG site were studied in a reference control population for all the probes: for each site, a reference methylation range was then established by considering the formula: upper limit=Q3+(3 x IQR) and lower limit=Q1-(3 x IQR) where Q1 is the first quartile, Q3 the third quartile and IQR the InterQuartile Range. Thus, for each site, epigenetic variations of each sample (including batch-matched controls, if provided) were identified as methylation values lying outside this interval (extreme outliers), classified as hyper-methylated or hypo-methylated by considering the median β-values of the reference cohort, and annotated as Stochastic Epigenetic Mutations (SEMs) in a new data matrix containing the burden of epigenetic variations and their genomic position. To detect the regions enriched in SEMs, an over-representation analysis of all identified SEMs was conducted by using a sliding window algorithm based on a cumulative hypergeometric distribution which tests the significant enrichment of SEMs in a window of a predefined size (e.g. 11 CpG sites) that slips (by single sites) on the annotated genome generating a window-associated p-value. If this p-value is below a predefined threshold, the algorithm keeps the central CpG site. The procedure is repeated in the adjacent windows generating a list of SEM-enriched regions. The function adopted to calculate SEMs is published atDOI: 10.5281/zenodo.3813234. Firstly, the method was applied to the reference population alone (n=68), then to the sample cohort (affected and unaffected twins). The β-value database of reference and samples cohorts was obtained by using the ChAMP (release 2.8.9) package [[9](#_ENREF_9)] in R environment (version 3.6.2). In the quality control and pre-processing stages, the sites with a detection p-value above 0.01 (n=6390) and a bead count <3 in at least 5% of samples (n=273) were discarded. Non-GpG probes (n=2929), potentially SNP affected probes (n=57525) [[10](#_ENREF_10)], probes aligning to multiple locations (n=11) [[11](#_ENREF_11)] and of X and Y chromosomes (n=9673) were also filtered out. As a final outcome of the pre-processing procedures, a total of 408711 CpG sites were retained and used for the analysis.

***Prioritization analysis of differentially methylated genes***

Gene ontology (GO) and pathway enrichment analyses were performed using GOorilla (<http://cbl-gorilla.cs.technion.ac.il/>) and Revigo (<http://revigo.irb.hr/>). The Phenolyzer software 20 (<https://phenolyzer.wglab.org/>) was queried using congenital hypothyroidism as disease terms.

**Statistics**

The “Shapiro.test” function provided in the R package “stats” was applied to test normality among variables. The “Wilcox.test” function provided in the R package “class” was used to test differences between cases and control groups for all non-parametric data. Considering the presence of categorical variables, dimensional reduction was performed using the Multiple Factor Analysis of Mixed data approach and the “FAMD” function provided in the R package “FactoMineR”. Linear mixed effect models and the lmer function provided in the R package lme4 v1.1-23 were adopted to assess differences in phenotypic traits and SEMs burden between affected and not affected twins. False Discover rate (FDR) correction was performed to correct for multiple testing.

***Data visualization***

Visualization of MultiDimensional Scaling and box-plots charts was produced by the “ggplot2” package in R.

***Supplemental methods references***

1. Caiulo S, Corbetta C, Di Frenna M, Medda E, De Angelis S, Rotondi D et al (2021) Newborn Screening for Congenital Hypothyroidism: the Benefit of Using Differential TSH Cutoffs in a 2-Screen Program. J Clin Endocrinol Metab. 106(1):e338-e49. **https://10.1210/clinem/dgaa789**

2. Medda E, Vigone MC, Cassio A, Calaciura F, Costa P, Weber G et al (2019) Neonatal Screening for Congenital Hypothyroidism: What Can We Learn From Discordant Twins? J Clin Endocrinol Metab. 104(12):5765-79. **https://10.1210/jc.2019-00900**

3. Wang K, Li M, Hakonarson H (2010) ANNOVAR: functional annotation of genetic variants from high-throughput sequencing data. Nucleic Acids Res. 38(16):e164. **https://10.1093/nar/gkq603**

4. Richards S, Aziz N, Bale S, Bick D, Das S, Gastier-Foster J et al (2015) Standards and guidelines for the interpretation of sequence variants: a joint consensus recommendation of the American College of Medical Genetics and Genomics and the Association for Molecular Pathology. Genet Med. 17(5):405-24. **https://10.1038/gim.2015.30**

5. Horvath S (2013) DNA methylation age of human tissues and cell types. Genome Biol. 14(10):R115. **https://10.1186/gb-2013-14-10-r115**

6. Pidsley R, CC YW, Volta M, Lunnon K, Mill J, Schalkwyk LC (2013) A data-driven approach to preprocessing Illumina 450K methylation array data. BMC Genomics. 14:293. **https://10.1186/1471-2164-14-293**

7. Leek JT, Storey JD (2007) Capturing heterogeneity in gene expression studies by surrogate variable analysis. PLoS Genet. 3(9):1724-35. **https://10.1371/journal.pgen.0030161**

8. Falcon S, Gentleman R (2007) Using GOstats to test gene lists for GO term association. Bioinformatics. 23(2):257-8. **https://10.1093/bioinformatics/btl567**

9. Morris TJ, Butcher LM, Feber A, Teschendorff AE, Chakravarthy AR, Wojdacz TK et al (2014) ChAMP: 450k Chip Analysis Methylation Pipeline. Bioinformatics. 30(3):428-30. **https://10.1093/bioinformatics/btt684**

10. Zhou W, Laird PW, Shen H (2017) Comprehensive characterization, annotation and innovative use of Infinium DNA methylation BeadChip probes. Nucleic Acids Res. 45(4):e22. **https://10.1093/nar/gkw967**

11. Nordlund J, Backlin CL, Wahlberg P, Busche S, Berglund EC, Eloranta ML et al (2013) Genome-wide signatures of differential DNA methylation in pediatric acute lymphoblastic leukemia. Genome Biol. 14(9):r105. **https://10.1186/gb-2013-14-9-r105**
