# Supplementary material for: Stochastic epigenetic mutations as possible explanation for phenotypical discordance among twins with congenital hypothyroidism
Source: J Endocrinol Invest. 2022 Sep 7;46(2):393–404. doi: 10.1007/s40618-022-01915-2 (PMC9859866; doi:10.1007/s40618-022-01915-2)
Supplement: Supplementary file 2 — Supplementary file2 (DOCX 8900 KB) [file 40618_2022_1915_MOESM2_ESM.docx]

**Supplemental Figure S1** Heatmap of the 450k array SNP probes (n=64). Samples with the same genetic background are expected to cluster together

**
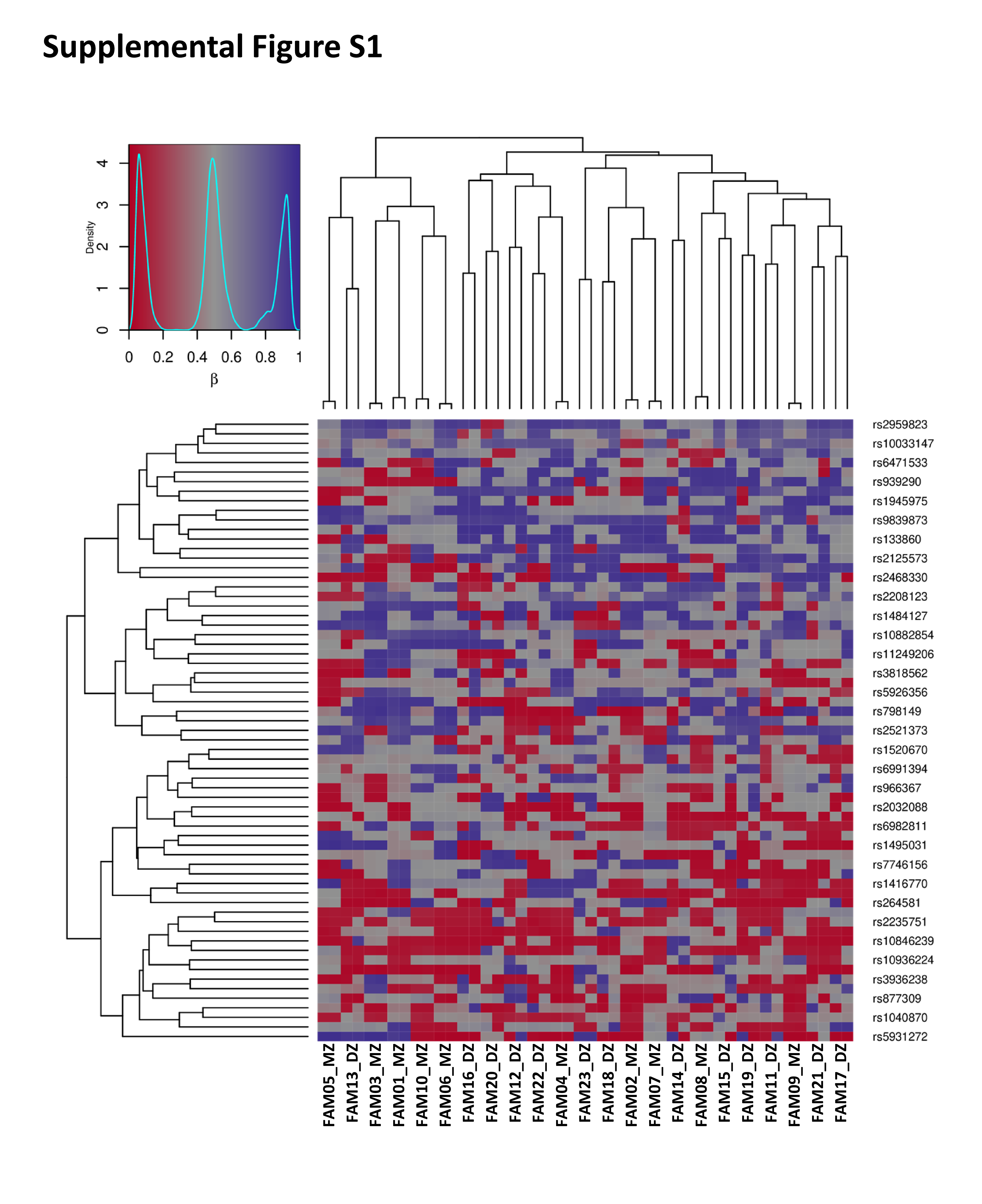
**

**Supplemental Figure S2** Boxplot showing the distribution of estimated cell counts between affected and unaffected groups. The thick horizontal line represents the median of the distribution while the box represents the interquartile range. Whiskers are set as the default option for boxplot function and extend to the most extreme data point which is no more than 1.5 times the interquartile range from the box. Open circles represent outliers (single values exceeding 1.5 interquartile ranges)

**
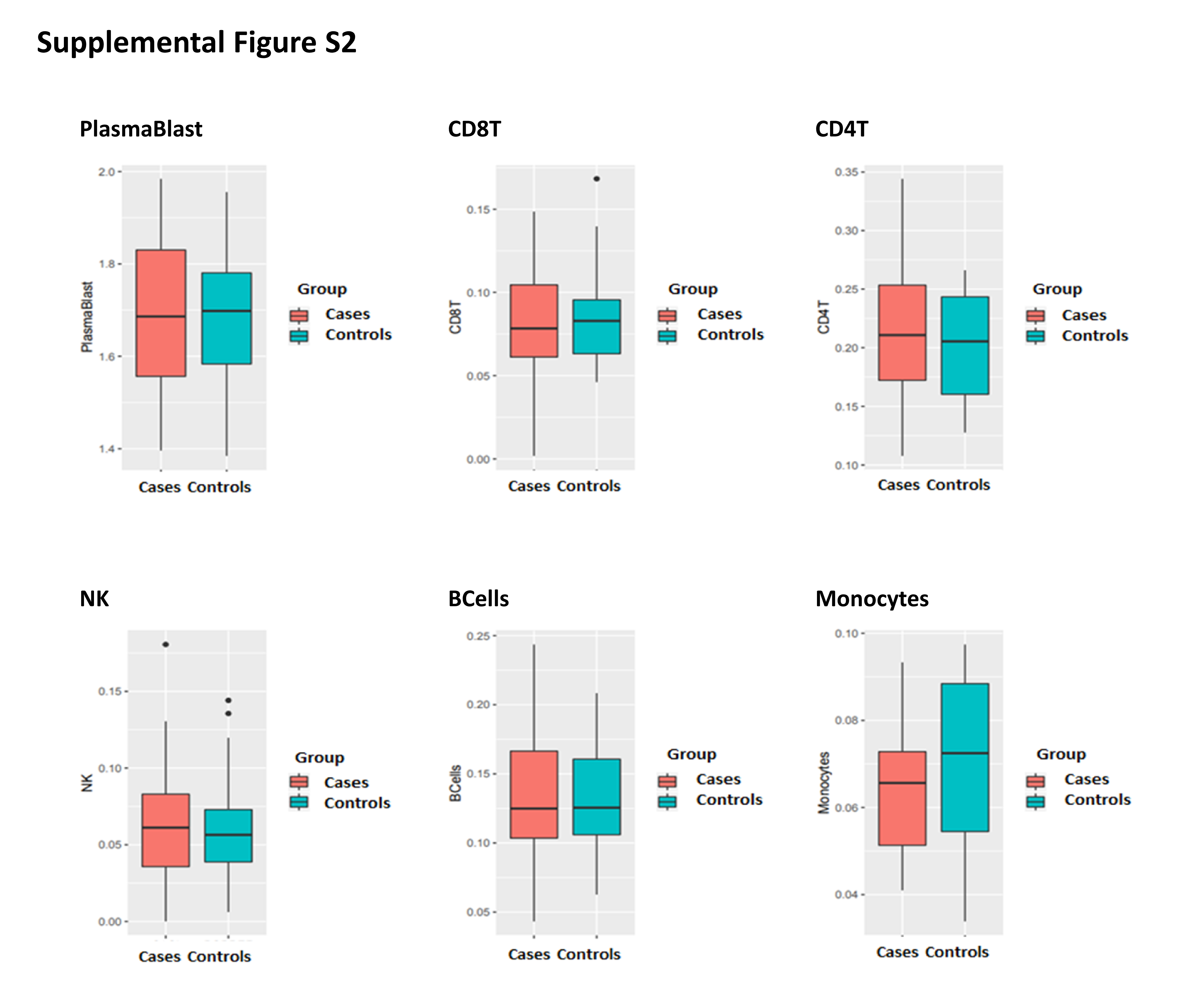
**

**Supplemental Figure S3** Scatter plot showing samples after performing Kruskal's non-metric multidimensional scaling. Only the first two dimensions are shown. Subjects are colored according to zygosity (a), presence of genetic variant (b), family (c) and presence of thyroid dysgenesis (d).


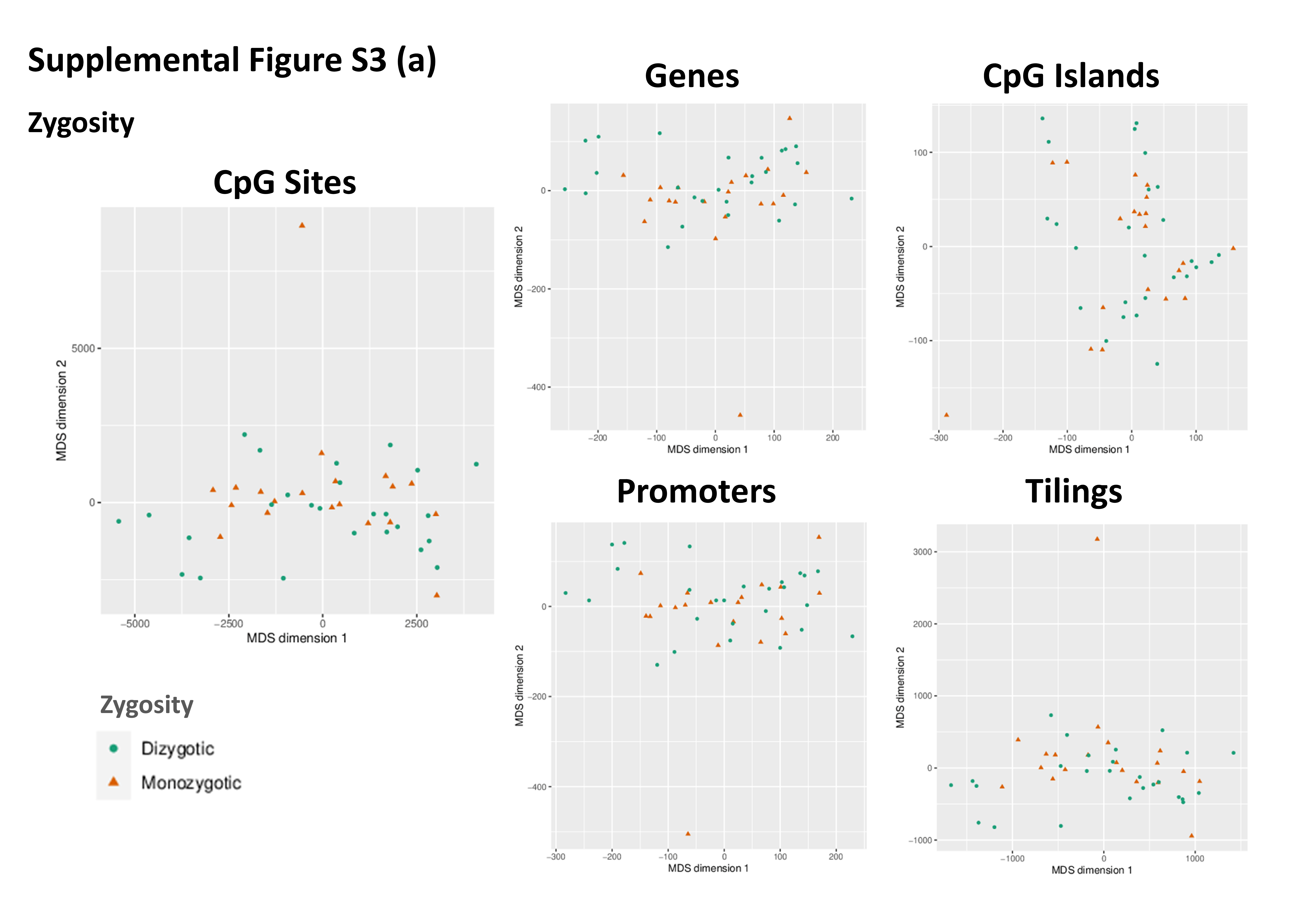


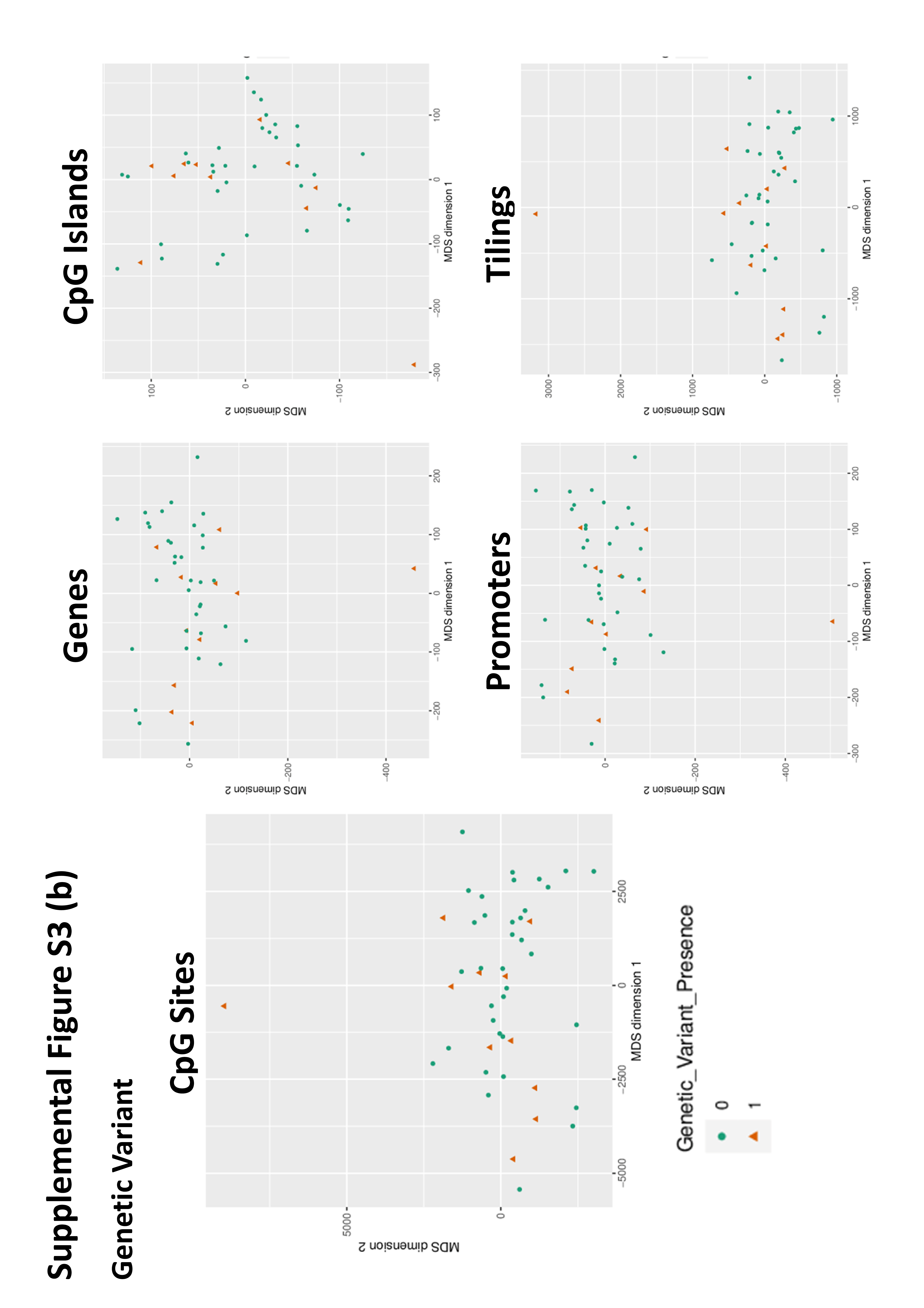


**
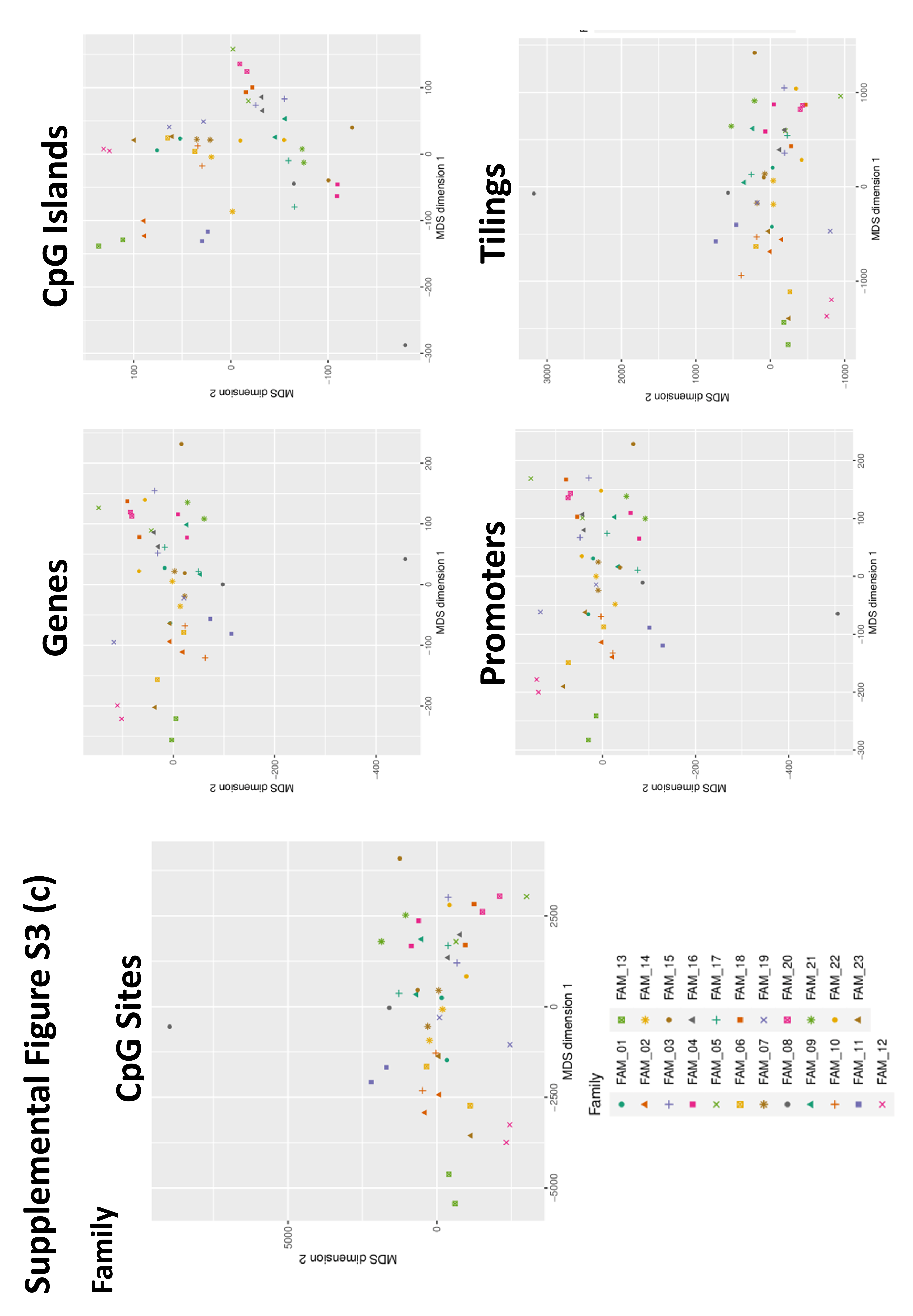
**

**
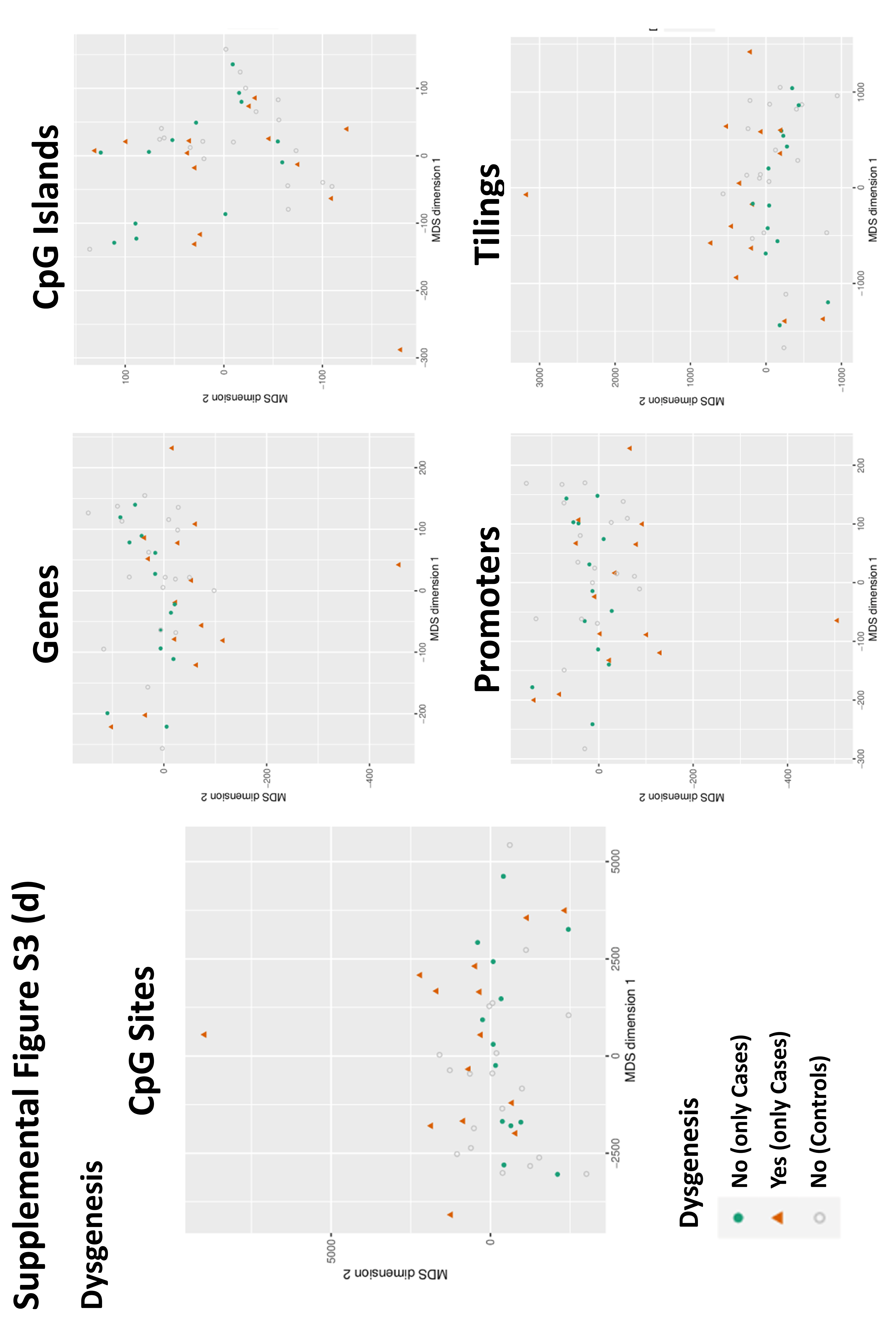
**

**Supplemental Figure S4** Gene ontology enrichment analysis performed on a subset of genes resulted significant at least at nominal level.


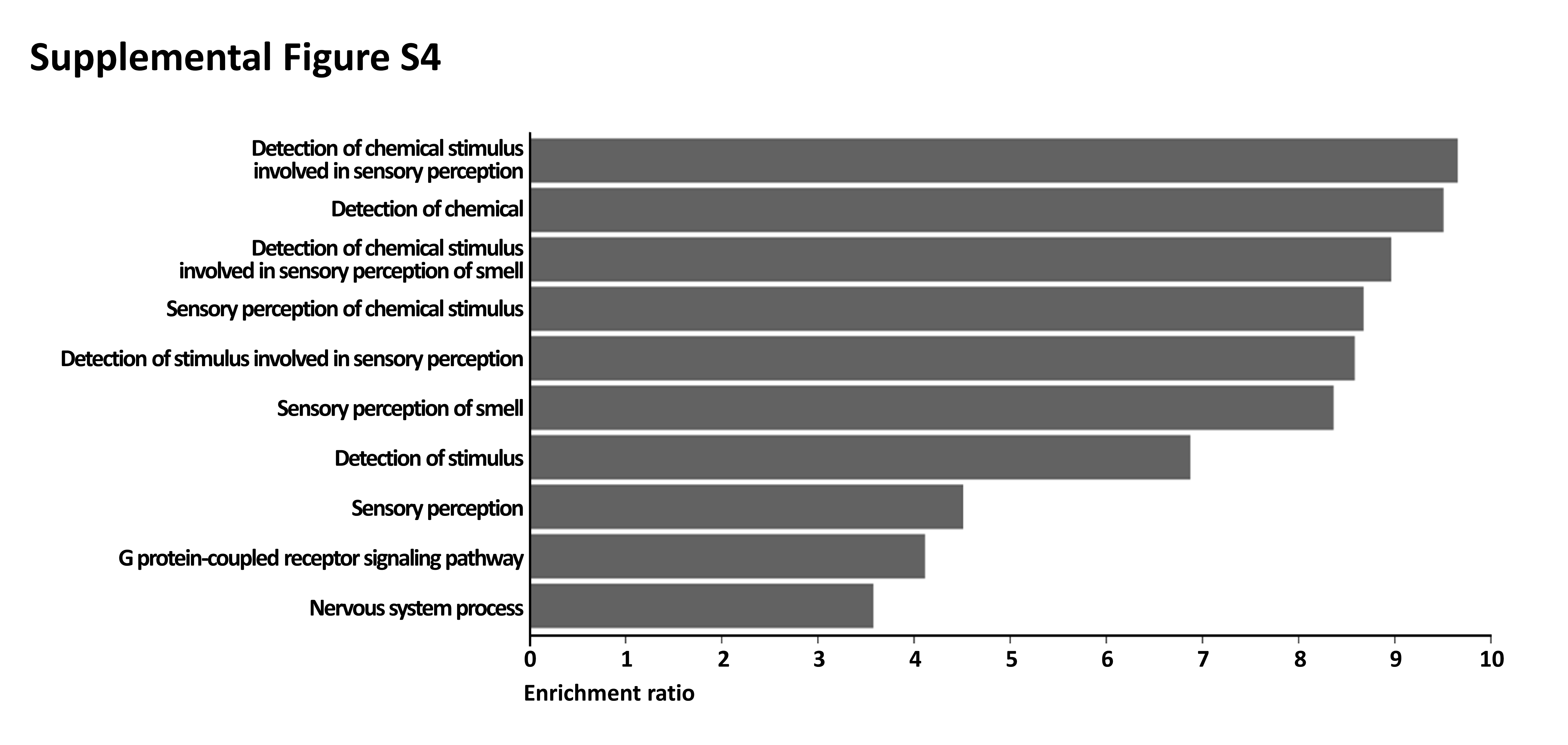


**Supplemental Figure S5** Revigo treemaps summarizing Gene Ontology (GO) analysis carried out on univocally epigenetically deregulated genes in case (CH) group: a) hyper-methylated genes, b) hypo-methylated genes.

**
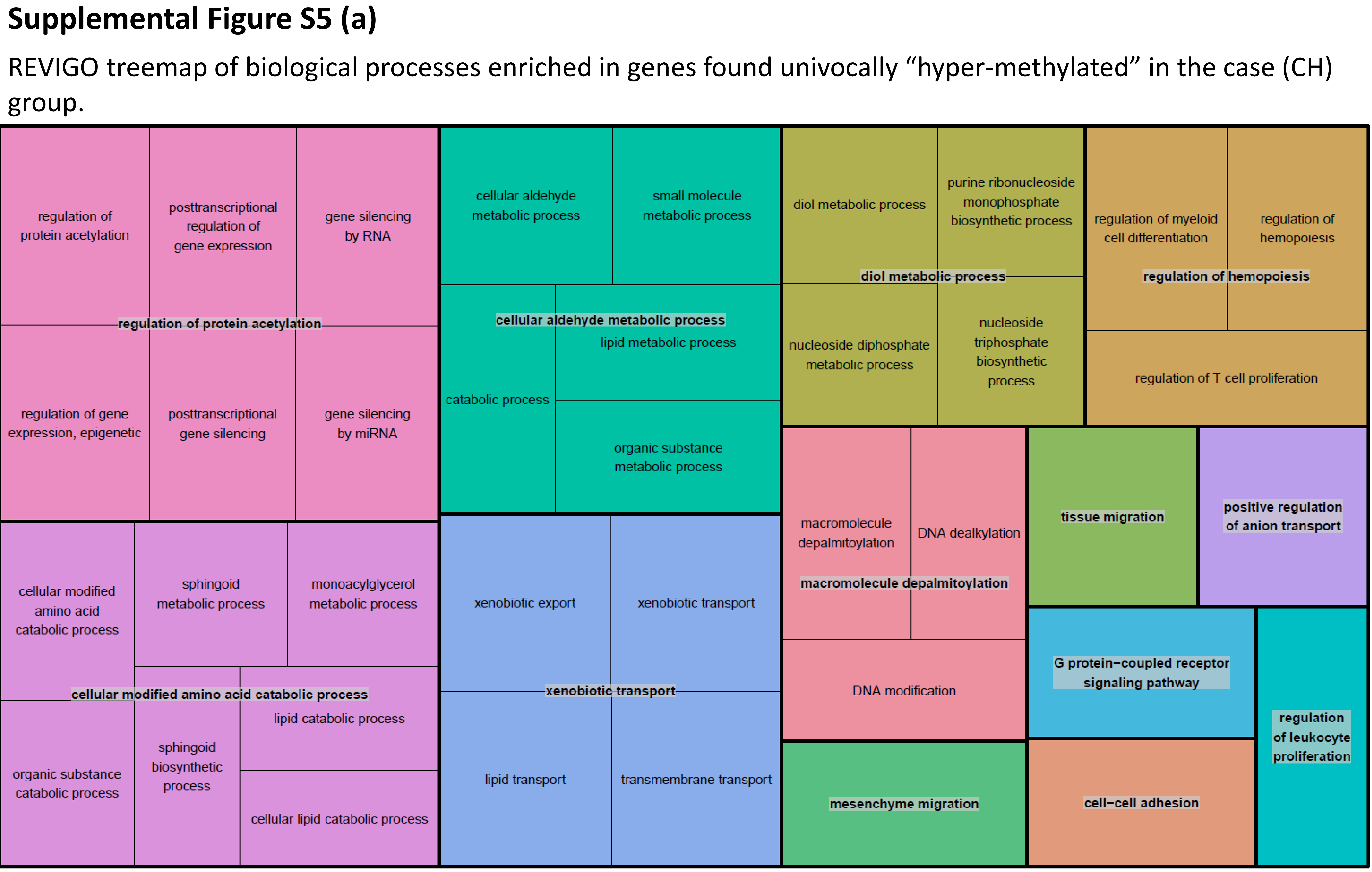
**

**
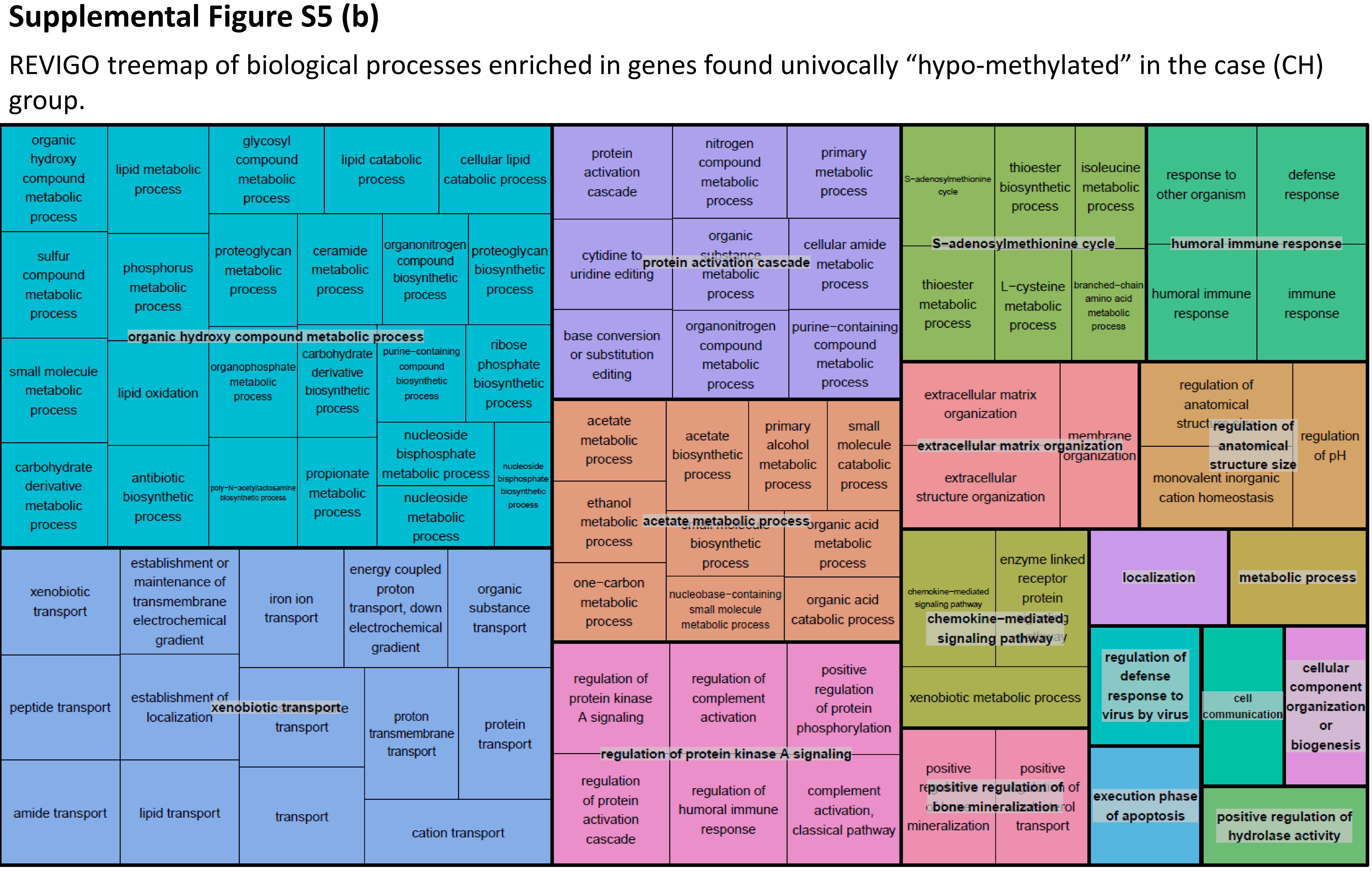
**
